# Protein Kinases in Alzheimer’s Disease: Pioneering Insights into Pathogenesis and Therapeutic Breakthroughs

**DOI:** 10.2174/011570159X379926250801062557

**Published:** 2025-10-08

**Authors:** Mohd Qasid Lari, Ajay Kumar, Astik Manju Ashesh, Deepak Kumar, Bhupendra Singh, Dileep Kumar

**Affiliations:** 1 Sardar Patel College of Pharmacy, Medical College Road, Gorakhpur, (U.P.), 273013, India;; 2 School of Pharmacy, Graphic Era Hill University, Dehradun, 248002, India;; 3 Department of Pharmacy, S.N. Medical College, Agra, 282002, India;; 4 Department of Pharmaceutical Chemistry, Manipal College of Pharmaceutical Sciences, Manipal Academy of Higher Education, Manipal, Karnataka, 576104, India

**Keywords:** Alzheimer’s disease, protein kinase, tau protein, amyloid precursor protein, GSK-3, CDK-5, neuroinflammation

## Abstract

Alzheimer's Disease (AD) is an exhausting neurodegenerative condition marked by the build-up of abnormal protein aggregates in the brain and a progressive loss of cognitive function. The complicated role that protein kinases play in the pathophysiology of AD has come to light more and more in recent years. The symptoms of AD include memory loss, cognitive impairment, and neuronal malfunction. Many cellular processes, including synaptic plasticity, neuronal survival, and protein homeostasis, have been linked to protein kinases, a class of enzymes that control phosphorylation. The etiology of AD has been closely related to the dysregulation of protein kinases, including those implicated in the phosphorylation of tau and the formation of amyloid-beta. GSK-3, also known as glycogen synthase kinase, is one of the most studied protein kinases in Alzheimer's disease. It is known that GSK-3 phosphorylates tau protein, causing it to clump together and create neurofibrillary tangles. Moreover, GSK-3 activation increases the development of amyloid-beta, which furthers the disease's progression. Additional protein kinases, including Cyclin-Dependent Kinase 5 (CDK5) and calcium/calmodulin-dependent protein kinase II (CaMKII), have also been connected to tau phosphorylation and synaptic dysfunction in AD. Protein kinases play a crucial role in the pathophysiology of AD, extending beyond tau phosphorylation. Research has shown that Amyloid Precursor Protein (APP) processing is regulated by Protein Kinases A (PKA) and C (PKC), which affects the production and clearance of amyloid-beta. Furthermore, AD etiology involves oxidative stress, neuroinflammation, and mitochondrial dysfunction, all of which are regulated by protein kinases. This study will cover the effects of protein kinases in AD, focusing on their role in tau phosphorylation, an attribute of the disease. We will also address the role of protein kinase in the development of amyloid-beta, synaptic malfunction, and neuroinflammation.

## 1 INTRODUCTION

The most prevalent neurodegenerative illness, Alzheimer's Disease (AD), was initially identified in 1907 by German physician Alois Alzheimer [1, 2]. The prevalence of Senile Plaques (SP) in the brain is one of the two main pathological alterations associated with AD. These misfolded proteins accumulate extracellularly, with β-amyloid (Aβ) being the primary constituent. Amyloid Precursor Protein (APP) is cleaved by β- and γ-secretase to provide the 36-43 amino acid peptide known as Aβ. The extracellular region of the protein is significantly removed when β-secretase truncates the N-terminus of APP. APP's residual C-terminus is subsequently cleaved to produce Aβ oligomers. After that, these oligomers come together to form aggregated plaques [3, 4]. Furthermore, Heat Shock Proteins (HSPs) like HSP90, HSP70, and HSP32 promote the synthesis of TNF-α and interleukin-6 (IL-6), which improves the removal of Aβ. It is known that Aβ can undergo various post-translational modifications. For example, pyroglutamate modification at GLU-3 can result in AβN3pE. As a result, N-terminally shortened Aβ is produced. Furthermore, phosphorylation at SER-8 can produce pSer8Aβ and pSer26Aβ [5]. There is a greater tendency for AβN3pE and pSer8Aβ to cluster into oligomers and fibrils [6]. On the other hand, pSer8Aβ and AβN3pE are only identified later in AD [7]. Therefore, the transition from preclinical to symptomatic AD may be caused by the formation of aggregates, the spread of deposits into additional brain areas, and the combined impact of elevated Aβ concentrations. Tau, a cytoskeletal protein that is abnormally hyperphosphorylated, is the main component of Neurofibrillary Tangles (NFTs), a notable pathological alteration. Within paired helical filaments, tau protein, which is connected to microtubules, is a crucial antigenic element [8]. The toxicity of neurons is primarily dependent on the accumulation of hyperphosphorylated Tau, which ultimately leads to the depolymerization of microtubules [9, 10]. Phosphorylated Tau protein aggregation can also lead to reduced dendritic spines, impaired synaptic transmission, and abnormal axonal transport [11]. Apart from impeding autophagy, this aggregation can also initiate a sequence of detrimental events, establish an adverse loop that escalates Tau protein aggregation, and eventually hinder cognitive processes related to memory and learning [11]. By removing cellular waste, recycling neurotransmitter molecules, and facilitating trans-synaptic transmission, astrocytes and microglia play a crucial role in maintaining neuronal function. Through the secretion of proteins, these glial cells mutually adjust their roles [12]. Nonetheless, astrocytes and microglia exhibit widespread activation in AD brains, resulting in the release of inflammatory mediators and a protective aggregation surrounding Aβ protein plaques. But this mechanism encourages neuropathology and neuroinflammation to proliferate [13].

Protein kinases mediate signal transduction and control a range of downstream consequences. They can phosphorylate other proteins to maintain their activity. They thus have a significant impact on the pathophysiology of AD. Here, we provide a thorough overview of the major protein kinases in AD, emphasizing their biological functions, implications, the most recent inhibitors, and substances that are about to enter the clinical stage.

## 2 METHODS

A comprehensive literature search was conducted to identify relevant research on protein kinases in Alzheimer's Disease, focusing on pioneering insights into pathogenesis and therapeutic breakthroughs. This search encompasses several key databases, including PubMed, Web of Science, ScienceDirect, Scopus, Cochrane Library, and Google Scholar.

### 2.1 Included and Excluded Criteria

This review included peer-reviewed studies (preclinical, clinical, and *in vitro*) specifically focused on Alzheimer’s disease, providing valuable insights into Pathogenesis and Therapeutic Breakthroughs. Studies were excluded if they were not peer-reviewed, reported irrelevant outcomes, were not published in English, or were duplicates.

## 3 CRUCIAL ROLE OF PROTEIN KINASES IN ALZHEIMER’S DISEASE

The pathogenesis of AD involves several kinases, including GSK-3β, p38 mitogen-activated protein kinase (p38 MAPK), CDK5, extracellular signal-regulated kinase-2 (ERK1/2), and c-Jun N-terminal kinase (JNK3). From Tau phosphorylation, Aβ accumulation, neurological inflammation, synaptic plasticity, and excessive stimulation of astrocytes and microglia, among other mechanisms, these kinases can facilitate the advancement of AD (Table **1**) [14-19].

## 4 IMPACT OF GLYCOGEN SYNTHASE KINASE-3-3 BETA (GSK-3Β) IN THE PATHOPHYSIOLOGY OF AD

Since its discovery, the only known function of GSK-3 has been to control glycogen metabolism. GSK-3's roles have been discovered to extend beyond glycogen control, as research has advanced. Apoptosis, cell differentiation, transcription, insulin action, circadian rhythm, and cell division are just a few of the biological processes now understood to be impacted by GSK-3 [20]. The hippocampus has a notably high concentration of GSK-3, which is broadly distributed throughout the central nervous system. The creation of memories and spatial navigation are largely dependent on the hippocampus, a part of the brain. It is essential to remember that GSK-3 has a complex and multifaceted role in various metabolic pathways and cellular activities. Research is still ongoing and is revealing new details about the roles and regulatory systems of GSK-3 [20].

### 4.1 GSK-3β Controls Tau Pathology

Most of the first studies on tau hyperphosphorylation focused on the kinase-phosphatase imbalance in the brain. They found that GSK3-β was a key tau kinase that could have a role in the development of AD tau pathology [21, 22]. The tau protein has a significant number of amino acid residues that are phosphorylated by GSK3-β [23]. Research on AD has also focused on the location of tau phosphorylation sites. Areas adjacent to microtubule-binding domains and associated amino acid residues exhibit tau protein phosphorylation driven by GSK3-β [24]. Since these binding domains are known to host protein-protein interactions, tau phosphorylation caused by GSK3-β is likely to lead to self-aggregation in a harmful manner [25]. A study employing a transgenic Drosophila model effectively revealed a positive correlation between the generation of hazardous tau aggregates and the abnormal overexpression of GSK3-β [24]. *In vitro* research further demonstrates that tau-filament clumps emerging from GSK3-β-induced phosphorylation in human neuroblastoma cells are strikingly comparable to AD tau pathology, which is consistent with the *in vivo* findings [26]. Because of this, as well as the fact that studies both *in vivo* and *in vitro* have demonstrated that GSK3-β inhibition causes tau to become hyperphosphorylated again, most drug research and discovery initiatives aimed at treating tau disease focus on GSK3-β activity [27].

### 4.2 GSK3-β Regulates Presenilin1 (PSEN1) Function

A significant contributing factor to Familial AD (FAD) has been confirmed to be the PSEN1 gene product, PRESENILIN 1 (PS1). PS1 is a transmembrane protein found in the cytosol or extracellular space. It consists of nine domains connected by hydrophilic loops. It contributes to the cleavage of APP and influences Notch signaling, the processing of β-catenin (not β-cadherin), and the metabolism of calcium [28]. Within the C-terminal transmembrane region of APP, PS1 facilitates the production of amyloid-β peptide (Aβ42) using γ-secretase following the cleavage of α- and β-secretase [29]. GSK-3β phosphorylates PS1 at serine residues (Ser353 and Ser357), which increases AD by inhibiting Aβ synthesis and APP cleavage while adjusting the Aβ 42/40 ratio [30]. Furthermore, PS1's ring structure domain facilitates the formation of a synaptic trimeric complex with N-cadherin and β-catenin. PS1's association with N-cadherin is similarly impacted by GSK-3β-mediated phosphorylation, which interferes with their binding and results in deficiencies in synaptic and neuronal activity [31]. The complex relationship between GSK-3β, PS1, and their effects on Aβ metabolism and synaptic function plays a significant role in the pathogenesis of AD.

### 4.3 GSK-3β Involvement in the Synaptic Plasticity Regulation

Synaptic plasticity is an initial sign of AD [32]. Long-Term Depression (LTD) and Long-Term Potentiation (LTP) are crucial factors in regulating the connections between neurons at the synapse [33]. Long-term Potentiation (LTP) is the term used to describe the process that opposes itself. In LTD, N-Methyl-D-Aspartate (NMDA) receptor channels on the postsynaptic membrane and alpha-amino-3-hydroxy-5-methyl-4-isoxazolepropionic acid (AMPA) play fundamental roles. The sodium-potassium cation channel AMPA helps to activate neurons, whereas the calcium ion channel NMDA does the same. A high-frequency stimulation brings about a substantial influx of calcium ions, and these ions bind with Calmodulin (CaM) to form Ca^2+^/CaM complexes. These complexes trigger LTP by activating CaMKII, a calcium- and calmodulin-dependent protein kinase. Excitation-to-pain, memory, learning, plasticity, and synaptic growth are all influenced by LTP [34]. A slight influx of calcium ions, triggered by low-frequency electrical stimulation, leads to LTD and AMPA phosphorylation [35]. The hippocampus, a critical brain region responsible for learning and memory, is the place where GSK-3β is most broadly expressed. There, it performs a critical regulatory role in maintaining a balance between LTP and LTD. The onset of LTD can be avoided by inducing LTP, and the induction of LTD is linked to a reduction in GSK-3β phosphorylation at Ser9. It is advantageous to inhibit GSK-3β in its active state to induce LTP [36]. Reduced LTP and anomalies in the formation of fear and spatial memories depending on the hippocampal region are observed in transgenic mice that overexpress the active form of GSK-3β. The GSK-3β inhibitor lithium can be given over an extended period to treat or prevent defective LTP [37].

**Fig. (1):**
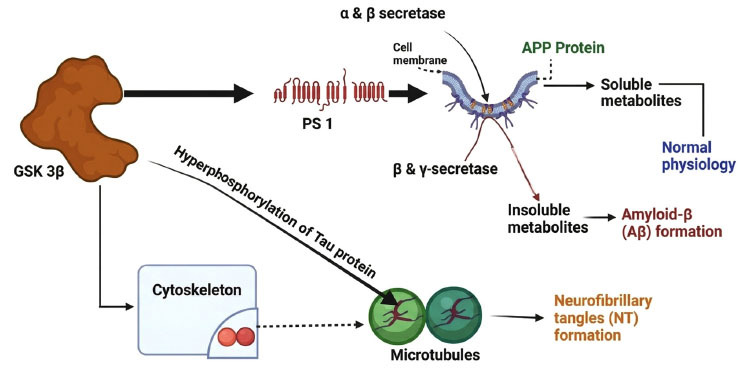
Action of GSK3 in regulating PS1, which leads to the formation of Amyloid beta and the hyperphosphorylation of Tau protein, resulting in the formation of neurofibrillary tangles and contributing to the pathogenesis of AD.

Thus, through various pathways, GSK-3β is a type of protein kinase that is essential to the pathophysiology of AD (Fig. **1**).

## 5 CYCLIN-DEPENDENT KINASE 5 (CDK5) IN THE PATHOPHYSIOLOGY OF AD

A crucial component of the CNS system, Cdk5 is involved in growth, maturation, and maintenance [38]. Cdk5 is a 33 kDa protein with 292 amino acids, comparable to other Cdk members in that they all possess catalytic cores with serine-threonine kinase activity. Nevertheless, Cdk5's activity is controlled by its association with the neuronal-specific activators p35 and p39, as well as their truncated versions, p25 and p29, rather than by its binding to cyclins [39].

The understanding of Cdk5's role in the pathophysiology of AD has advanced significantly in recent years. An increasing amount of data suggests that Cdk5 dysregulation influences several intracellular signaling pathways, which in turn promote the neurodegenerative pathogenesis of AD. It has been shown that Cdk5 causes intracellular accumulations of hyperphosphorylated tau in NFTs and extracellular deposition of Aβ in senile plaques. Furthermore, an abundance of evidence suggests that the subsequent improper gain-of-function of Cdk5 is also linked to neuronal cell death, synaptic abnormalities, mitochondrial dysfunction, and cell cycle activation, all of which repeatedly contribute to the pathophysiology of AD by interacting with tau and Aβ.

### 5.1 Cdk5 Promotes Aβ Formation

The major component of amyloid plaques, amyloid-β peptide, accumulates over time and is a significant early trigger in the development of AD [40]. Aβ in the amyloidogenic pathway is produced by β-secretase (BACE1) cleaving the integral membrane glycoprotein APP sequentially, followed by γ-secretase cleavage in the transmembrane region, which releases Aβ peptides [41]. A few mutations in the Aβ synthesis-related genes APP, PSEN1, Mol Neurobiol, and PSEN2 can cause familial AD [42]. The close relationship and reciprocal effects between Cdk5 and Aβ result in a vicious cycle. The production of Aβ and its accumulation in the cell body and neurite are closely linked to Cdk5. This leads to neurotoxicity, which is linked to a sequence of pathological events known as the amyloid cascade, including synaptic damage, neuronal dysfunction, hyperactivation of kinases, and ultimately, neuronal loss [43]. In transgenic mice overexpressing p25, pharmacological suppression of Cdk5 activity decreases Aβ synthesis and attenuates Aβ-induced neuronal death. Additionally, phosphorylating APP has been linked to Cdk5, increasing its susceptibility to the production of Aβ. Numerous studies have shown that Cdk5 is a substrate of APP and that Cdk5 has documented phosphorylation at Thr668 [44]. p25 transgenic mice have been shown to exhibit elevated APP Thr668 phosphorylation, increased generation, and overaccumulation of Aβ compared to wild-type mice [45]. Cdk5 can phosphorylate APP at Thr668 to regulate its processing, increase Aβ production, and reduce APP's ability to interact with the cytoplasmic adaptor protein Fe65, thereby preventing Aβ formation. Thr668 phosphorylation of APP also affects APP's endocytic trafficking, which aids in APP's β-secretase cleavage and boosts the production of Aβ. The phosphorylation of APP by Cdk5 can also alter the activity of other kinases, like GSK-3β, allowing APP to regulate Aβ generation. A connection exists between changes in the presenilin system and the deregulation of Cdk5. *In vitro* and *in vivo* experiments demonstrate that CDK5 can increase presenilin levels by directly phosphorylating PS1 at Thr354, which is essential for γ-secretase activity and Aβ catabolism [46]. Additional pathogenic pathways exist whereby Cdk5 facilitates the synthesis and build-up of Aβ. Cdk5 upregulates the phosphorylation of STAT3, enhancing BACE1 transcription, which in turn increases the amyloidogenic processing of APP to produce Aβ. Through NGF deprivation, Cdk5 causes neuronal death, which is followed by the phosphorylation of tropomyosin-related kinase A (TrkA). This process is reliant on the production of Aβ and the activities of β- and γ-secretase [47]. Nevertheless, peptides raise intraneuronal calcium concentrations, which in turn trigger calpain activation, cleave p35 to p25, and ultimately lead to dysregulation of CDK5 activity [48]. In summary, Cdk5/p25 regulates APP metabolism, resulting in abnormal phosphorylation of APP, which affects the production of Aβ. On the other hand, Aβ might be involved in the aberrant Cdk5 activity. As a result, Cdk5 and Aβ work together to create a positive feedback loop that sets off a series of pathogenic AD events. Since Cdk5 is linked to the phosphorylation of AP, accumulating evidence has indicated that aberrant activation of Cdk5 plays a role in the early stages of AD. Therefore, blocking Cdk5 hyperactivity may prevent the death of neurons caused by Aβ buildup and halt AD progression.

### 5.2 Cdk5 Impact on Tau Phosphorylation

**Fig. (2):**
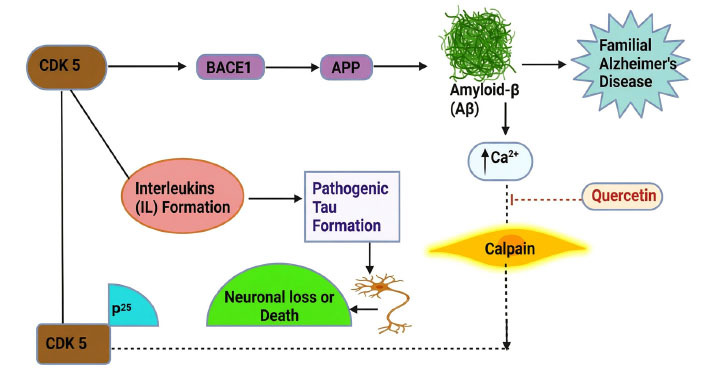
Impact of CDK5 in the pathogenesis of AD through Aβ formation and tau phosphorylation, and the effect of quercetin in the management of this disease by inhibiting the Ca^2+^ calpain pathway.

A prominent feature of AD pathogenesis is the development of neurofibrillary tangles in neurons, accompanied by tau phosphorylation. Neuronal apoptosis results from the aberrant phosphorylation and deposition of tau, a cellular microtubule-related protein, which alters microtubule function and disrupts the cytoskeleton structure. Tau is a substrate for several kinases, including PKA, GSK-3β, and Cdk5. Cdk5 is essential for the phosphorylation of tau and the development of neurofibrillary tangles [49]. A necessary route for tau hyperphosphorylation in AD is the miR-148a-3p/p35/PTEN signaling pathway [50]. Numerous elements, including RPS23RG1 [51] and MARK4 [52], are linked to tan disease by regulating Cdk5 activity. Glutamate triggers the transcription of p35 and Cdk5 mRNA. Tau hyperphosphorylation is a result of the Cdk5/p25 complex's glutamate-responsive increase [53]. As discussed earlier, AD inflammation is also linked to Cdk5 [54]. Through the Cdk5 pathway, the inflammatory agent leukotriene may hasten the pathogenic buildup of tau. In addition to alleviating behavioural abnormalities and preventing neuronal death, Cdk5 Inhibitory Peptide (CIP) can lower inflammation and tau hyperphosphorylation [55]. Quercetin and other medications can block the tau pathogenic process by inhibiting the Ca^2+^-calpain-p25-Cdk5 pathway [56] (Fig. **2**). Given its critical function in both tau phosphorylation and neurofibrillary tangle development, Cdk5 is considered a promising target for AD therapy.

### 5.3 Electing Mitochondrial Dysfunction *via* Cdk5

Numerous investigations have demonstrated that mitochondrial dysfunction and oxidative stress occur rapidly in the pathology of AD before the appearance of observable plaques and tangles [57]. Cdk5 plays a direct role in the production of Aβ and glutamate, two key molecules in AD pathogenesis, as well as contributing to oxidative stress and mitochondrial abnormalities in neurons.

According to reports, in post-mitotic neurons, Cdk5 negatively phosphorylates Dynamin-related protein 1 (Drp1) at serine 616. This is important for changing the morphology of the mitochondria, which is implicated in numerous cellular physiologies and diseases [58]. Drp1 is drawn to the Mitochondrial Outer Membrane (MOM) from the cytosol. There, it forms ring-shaped structures that encircle the MOM and pierce the membrane after GTP hydrolysis [59]. When Drp1 is phosphorylated at S616 by Cdk5, it causes an increase in GTPase activity and mitochondrial translocation, which in turn accelerates mitochondrial fission under pathological conditions [60]. Neuronal death and mitochondrial abnormalities are linked to excessive mitochondrial fission. Therefore, in illness models, pharmacological or genetic suppression of Cdk5 attenuates Drp1-induced mitochondrial fission, restoring mitochondrial ATP production and conferring neuroprotection [61].

## 6 c-JUN N-TERMINAL KINASE-3 (JNK-3) ASSOCIATED WITH THE PATHOGENESIS OF ALZHEIMER’S DISEASE

JNK has been the focus of intense scientific attention since its discovery over 20 years ago. Efforts are still being made to assess its biochemistry and regulation, as well as its role in both physiological and pathological cellular activities. There are three families of mitogen-Activated Protein (MAP) kinases, and the JNK family is one of them. Jnk1 (MAPK8), Jnk2 (MAPK9), and Jnk3 (MAPK10) are three genes that encode ten distinct isoforms with molecular weights ranging from 46 to 55 kDa. JNK3 is primarily found in neurons and is also present in the heart and testis to a lesser extent, whereas JNK1 and JNK2 are distributed across a wide range of tissues [62]. Human postmortem brain tissues from AD patients have shown positive co-localization with Aβ and increased production of phosphorylated JNK (pJNK) [63]. JNK3, in particular, is highly expressed and activated in the cerebral tissue and brain fluid of AD patients, showing a statistically significant correlation with the rate of cognitive deterioration [64]. Aβ peptides have been shown to activate JNK *in vitro*, as demonstrated by experiments on C57BL/6 mouse primary cortical and hippocampus cultures, Wistar rat primary cortical cell cultures, and SH-SY5Y neuroblastoma cells, which reveal an increase in p-JNK after Aβ treatment [65]. Interestingly, research has revealed that JNK3 is the primary kinase responsible for phosphorylating the β-Amyloid Precursor Protein (APP) at T668. In fact, genetic JNK3 knockdown significantly reduced Aβ42 peptide levels and overall plaque loads in transgenic AD mice, while also increasing neuronal density and improving cognition [66]. Research has confirmed that APP cleavage is regulated by JNK3-mediated phosphorylation, leading to the amyloidogenic processing of the protein. Conversely, JNK suppression *in vitro* stimulates the non-amyloidogenic route by inhibiting APP phosphorylation [67].

In experimental models of AD, JNK activation is associated with increased levels of senile plaques and NFTs, according to research using a mouse model of AD that includes the Swedish APP mutation with a mutant PS1 (Tg2576/PS1) [68]. Additionally, studies using experimental models of AD that are based on established risk factors for the onset of AD, such as stress or insulin resistance, have been carried out [69]. JNK phosphorylation is elevated in mice exposed to chronic moderate stress (CMS), which is known to enhance tau misprocessing and amyloidogenic processing [70]. Subdiabetogenic doses of Streptozotocin (STZ) injected intracerebroventricularly resulted in elevated levels of pJNK, oxidative stress, insulin resistance, and abnormalities in cognition and brain cholinergic function [71-73]. It should be emphasized that because JNK phosphorylates Insulin Receptor Substrate (IRS) 1, it can potentially directly cause insulin resistance by obstructing the transduction signal that the insulin receptor produces [74].

Additional potential roles for c-Jun in AD have been discovered. For example, phosphorylation of c-Jun within the structure of NFTs may indirectly govern the maturation of tangles in AD, which is primarily regulated by JNK phosphorylation of c-Jun. Because of the imbalance between the phosphorylation of c-Jun by JNK and the reduced synthesis of PP2A (protein phosphatase 2), phosphorylated c-Jun is more prevalent than non-phosphorylated c-Jun. Phosphoryl-c-Jun shows a lower tendency to be broken down by proteasomes, which leads to its accumulation inside NFTs and facilitates the formation of tangles [75].

Through the direct phosphorylation of Tau, c-Jun N-terminal kinase also directly regulates the production of NFTs [76]. *In vitro* phosphorylation experiments have demonstrated that the JNK3 isoform may substantially auto-phosphorylate itself and contribute to Tau hyperphosphorylation [77]. It has been found that JNK phosphorylates Tau at Ser422. More specifically, JNK3 has the highest affinity for phosphorylation at Ser422, which controls caspase-3's hydrolysis of Asp421. Indeed, it has been demonstrated that phosphorylation at Ser422 guards against caspase hydrolysis at Asp421. Under normal conditions, tau is responsible for numerous intra- and extracellular signaling mechanisms, in addition to stabilizing the neuronal cytoskeleton by binding to tubulin monomers [78-79].

## 7 THE FUNCTION OF PROTEIN PHOSPHATASES IN PRESERVING KINASE HOMEOSTASIS

A dynamic and reversible process, protein phosphorylation is crucial for regulating various cellular processes, including signal transduction, synaptic plasticity, and cell viability. Protein kinases, which add phosphate groups, and protein phosphatases, which remove them, work in tandem to closely regulate this process. The development of neurodegenerative diseases, including AD, is closely linked to an imbalance between these conflicting activities, which can result in abnormal signaling [80].

Protein Phosphatase 2A (PP2A), one of the serine/threonine phosphatases, has become a key factor in neuronal function due to its essential role in dephosphorylating tau protein. Tau must remain dephosphorylated for PP2A to perform its regular job of maintaining microtubules in healthy neurons. However, tau hyperphosphorylation, microtubule destabilization, and the formation of neurofibrillary tangles result from the markedly decreased PP2A activity in AD, which can be caused by altered expression, post-translational modifications, or the presence of endogenous inhibitors, such as the SET protein [81].

According to recent research, PP2A activity restoration may have neuroprotective benefits. For instance, in animal models, sodium selenate, a well-known PP2A activator, has been shown to lessen tau pathology and cognitive impairment. The translational significance of targeting phosphatases in therapeutic techniques was further supported by a recent Phase 2a clinical trial that showed its safety and potential to delay neurodegeneration in early AD patients [82]. Additionally, PP2A modulates synaptic transmission, inflammatory signaling, and APP processing—processes that are often dysregulated in AD. Its wide range of regulation highlights the potential advantages of phosphatase-targeted therapies in reducing related pathogenic cascades as well as in reversing aberrant phosphorylation states [83].

Enhancing phosphatase activity provides a natural counterbalance, as kinases such as GSK-3β and CDK5 are overactive in AD and contribute to the progression of the illness by excessively phosphorylating tau and other substrates. Restoring phosphatase function may lead to therapeutic outcomes with better selectivity and fewer side effects than solely blocking kinases, which often cause off-target effects and systemic toxicity.

## 8 TREATMENT FOR ALZHEIMER'S DISEASE WITH PROTEIN KINASE INHIBITORS

Owing to the complex role that protein kinases play in the etiology of AD, scientists have worked hard to create inhibitors of GSK-3β, p38 MAPK, CDK5, and JNK3 for AD. This section focuses on the use of these kinase inhibitors in the treatment of AD (Table **2**) [84-93].

### 8.1 GSK3β as a Medicinal Strategy

Since the formation of Aβ, tau phosphorylation, neurogenesis, or memory, and synaptic dysfunction have all been definitively linked to GSK-3β signaling as one of the neuropathological features of AD, blocking this signaling has become a potentially effective therapeutic strategy for treating AD. Numerous pathologic symptoms linked with AD have been effectively addressed by loss-of-function research using a variety of AD mouse models [94]. It has been shown that the mood-stabilizing drug lithium reduces levels of Aβ and phospho-tau in several animal models of Alzheimer's [91-92]. Tideglusib, a GSK-3β inhibitor, enhanced memory deficits in APP by reducing tau phosphorylation, Aβ deposition, and astrocyte proliferation. Overexpressing human mutant APP in sw-tauvlw mice [93, 94]. Ultimately, the positive impact of lowering GSK3-β activity in an AD model was validated by brain infusion of the particular GSK3 inhibitor SB216763 [95].

Despite being a highly conserved kinase, only a few numbers of GSK-3 inhibitors that were previously evaluated in preclinical stages have advanced to clinical trials. Furthermore, despite a few positive findings in animal models, the widespread expression of GSK3-β and its involvement in several crucial cellular biological activities have created numerous concerns and significant toxicological obstacles for chronic therapy studies in both the pre-clinical and clinical phases [96, 97]. AZD2558 was the first contender for a phase I clinical trial as a GSK3-β inhibitor. Both *in vitro* and *in vivo*, this highly selective GSK3-β inhibitor effectively decreased tau phosphorylation and gliosis. However, testing of this medication for the long-term treatment of AD was impeded by the severity of off-target effects linked to its *in vivo* delivery. An additional attempt was made with AZD1080, which has been shown *in vitro* and in preclinical research to be capable of reducing tau phosphorylation. Regrettably, the phase I trial was stopped because long-term use of this medication was also linked to serious adverse effects [98]. As of right now, Tideglusib is the only GSK-3 inhibitor approved for use in phase II clinical trials for the treatment of AD and progressive supranuclear palsy. Two modest Phase II clinical trials involving people with mild to moderate AD examined the efficacy of tideglusib. The results revealed good tolerability but no appreciable clinical changes [99]. Patients with a clinical diagnosis of AD and mild cognitive impairment have participated in pilot clinical trials using lithium chloride. One FDA-approved medication for bipolar disorders is lithium chloride, which is a poor and non-specific GSK3-β inhibitor. It can decrease GSK3-β activity by around 25% at therapeutic levels without having any adverse side effects [100]. Minimal clinical trials including this substance have demonstrated some encouraging outcomes, such as enhanced cognitive function and decreased tau phosphorylation, indicating that lithium may have value as an AD disease-modifying treatment [96].

### 8.2 Cdk5 Inhibiting Drugs used in Alzheimer's Disease

To date, none of the specific medications that directly target CDK5 for the treatment of AD have received approval for clinical use; these are currently in the experimental stages of development. The kinase CDK5 is involved in various cellular functions, including synaptic plasticity and brain development. However, deregulation of this enzyme has been linked to neurodegenerative illnesses, including Alzheimer's. Research has focused on identifying small chemicals and substances that can specifically inhibit CDK5 activity, aiming to alleviate aberrant tau phosphorylation and synaptic dysfunction associated with Alzheimer's disease.

In a recent study, TgCRND8 mice were administered Sulforaphane (SF) intragastrically at doses of 25 and 50 mg/kg for four months, starting at three months of age. The cognitive abilities were assessed using the Morris Water Maze Test. Aβ1-42 oligomers were co-treated after SF pretreatment of cultured primary mouse neurons. To determine the role of the CDK5/p25 pathway in the anti-AD effects of SF in primary neurons, roscovitine, a CDK5 inhibitor, was used. The findings demonstrated that SF therapy prevented Aβ1-42-induced neurotoxicity in primary mouse neurons and markedly improved cognitive abnormalities in TgCRND8 animals. In TgCRND8 mice, SF may decrease tau protein phosphorylation at specific locations and modify the expression of markers associated with Aβ generation. Furthermore, SF upregulated the expression of CDK5 and markers related to synaptic plasticity. Additionally, SF significantly reduced CDK5/p25 activity [101]. Thus, it was concluded that Strong CDK5 inhibitor SF has the potential to be used as a therapeutic drug to treat and prevent AD. Furthermore, SF prevented the overexpression of CDK5 in mouse primary neurons [101].

Another study on Roscovitine and its derivatives suggests that roscovitine derivatives can inhibit cdk5 and exhibit fewer side effects and greater efficacy than roscovitine [102]. The cyclin-dependent kinase inhibitor roscovitine, also known as seliciclib, has been investigated for its ability to inhibit CDK5. Preclinical research has demonstrated its potential in lowering tau phosphorylation [102]. The purine analog inhibitor roscovitine, which has a broad spectrum of action, inhibits CDK1, CDK2, CDK5, CDK7, and CDK9, but not CDK4, CDK6, or CDK8 [103]. It is effective in reducing tau phosphorylation, cell cycle entrance, DDR, and neuronal death, even if it is not as selective for CDK5. Because roscovitine is non-specific with CDK5, it reduces a variety of abnormalities but is hazardous to tissues that are not its target [104, 105].

### 8.3 Targeted Therapy for Inhibiting JNK-3

To treat Alzheimer's disease, research is still being done on developing drugs that specifically target c-Jun N-terminal Kinase 3 (JNK3). JNK3, a member of the MAPK family, plays a crucial role in various biological processes, including apoptosis and inflammation. JNK3 dysregulation has been connected to neurodegenerative diseases, including Alzheimer's disease. A pan-JNK inhibitor, SP600125 (PubChem ID 8515), was among the first to be studied. For JNK1, JNK2, and JNK3, their IC50 values were 40, 40, and 90 nM, respectively. It has been demonstrated to prevent the production of βAPP, which results in neuronal death, in both *in vitro* and *in vivo* models of AD. Furthermore, prior research indicates that intracerebroventricular infusions of SP600125 enhanced AD-related neurological traits in animal models. However, this type of molecule exhibits relatively low selectivity. The lack of specificity of SP600125 results in varying responses and toxic profiles, despite its continued use in research. Still, it advances our knowledge of JNK's involvement in several physiological and pathological conditions [106, 107].

A pan-JNK inhibitor, Tanzisertib (Celgene) (PubChem ID 11597537), was created by modifying the SP600125 structure and has been shown to block JNK3 activity [108]. This compound, also known as CC-930, was the first to be discovered orally and was being investigated in phase II clinical trials to treat idiopathic pulmonary fibrosis (NCT01203943) and discoid lupus erythematosus (NCT01466725). The studies were terminated in 2012 due to the occurrence of substantial hepatotoxicity [109].

## 9 TOXICOLOGICAL CONSIDERATIONS OF PROTEIN KINASE INHIBITORS

Although targeting protein kinases in Alzheimer's disease has promise, their clinical translation has been hampered by serious toxicological concerns. Due to their widespread physiological responsibilities, specific kinases, such as GSK-3β, CDK5, and JNK3, regulate processes outside the central nervous system, including immunological responses, cell division, and metabolism [110].

For example, although Tideglusib (a GSK-3β inhibitor) reached Phase II clinical trials, its results showed no cognitive improvement, sparking concerns about long-term toxicity due to GSK-3β's widespread involvement in various organs [111]. Similarly, despite initial neuroprotective promise, Tanzisertib (a pan-JNK inhibitor) was stopped during trials for other inflammatory illnesses because of hepatotoxicity [20].

Although roscovitine (seliciclib), a CDK5 inhibitor, has demonstrated effectiveness in lowering tau phosphorylation, its poor selectivity (targeting CDK1, 2, 5, 7, and 9) has led to off-target effects, such as cytotoxicity in non-neuronal tissues [112]. Furthermore, many kinase inhibitors have poor BBB permeability, which requires a higher systemic dose and raises the possibility of side effects [113].

To improve BBB penetration while reducing peripheral toxicity, future methods should prioritize CNS-selective agents, prodrugs, or delivery systems based on nanoparticles. Additionally, inducible or short-term inhibition might offer safer substitutes for long-term systemic exposure.

## 10 STUDY LIMITATIONS

This review is based on currently available preclinical and clinical evidence regarding protein kinases in Alzheimer’s disease. However, the heterogeneity of study models, variability in drug responses, and limited number of long-term human trials may constrain the generalizability of these findings. Additionally, the lack of standardized biomarkers for assessing kinase activity *in vivo* poses a challenge in correlating mechanistic data with clinical outcomes.

## CONCLUSION

Several complex and dynamic molecular pathways, including protein kinases, are involved in the pathophysiology of AD. Several kinases, including cyclin-dependent kinase 5 (CDK5), c-Jun N-terminal kinase (JNK), and glycogen synthase kinase-3 beta (GSK-3β), are essential for the abnormal phosphorylation of tau, which is a significant factor in the production of neurofibrillary tangles, a characteristic of AD. Apart from tau pathology, these kinases also play a role in the buildup of amyloid-beta (Aβ), oxidative stress, mitochondrial dysfunction, and chronic neuroinflammation, all of which contribute to the death of many neurons and cognitive impairment. These kinases function as part of a larger signaling network that regulates vital biological functions such as neurotransmission, synaptic plasticity, and neuronal survival. The development of the disease is exacerbated by dysregulation in this kinase signaling, which not only accelerates neurodegeneration but also alters glial activation and compromises the blood-brain barrier. The diverse functions of protein kinases in AD highlight their potential as highly effective treatment targets.

Fortunately, there is increasing preclinical evidence that selective kinase inhibitors can improve AD pathogenesis. In various *in vitro* and *in vivo* settings, inhibitors targeting GSK-3β, CDK5, JNK3, and other kinases have demonstrated effectiveness in reducing tau phosphorylation, inhibiting Aβ accumulation, and improving synaptic function and memory. However, various obstacles have been in the way of turning these discoveries into successful human medicines. Because kinase enzymes have a wide range of physiological functions, many kinase inhibitors have systemic toxicity, off-target effects, and limited blood-brain barrier penetration. Clinical progress is further complicated by the variability in patient response and the absence of accurate biomarkers for disease monitoring. Future approaches should prioritize the development of highly selective, brain-penetrant kinase inhibitors with low peripheral toxicity to overcome these obstacles. Allosteric modulators, prodrug strategies, and nanocarrier-based delivery systems may provide viable solutions. A systems biology method that combines artificial intelligence and multi-omics data can also be used to identify new kinase networks and patient-specific targets. Restoring kinase-phosphatase equilibrium is also crucial and should not be disregarded. Treatments that simultaneously increase phosphatase activity, especially PP2A, may modify illness in a more physiological and long-lasting way. Combination therapy regimens that include protein kinase modulation, possibly in conjunction with tau aggregation inhibitors or anti-Aβ antibodies, may further enhance therapeutic outcomes.

In conclusion, protein kinases are a crucial mechanistic element and a potentially effective treatment option in the context of Alzheimer's disease. To turn existing knowledge into disease-modifying treatments, researchers, physicians, and pharmaceutical developers must continue their interdisciplinary partnership. There is cautious hope that modulating kinase activity may soon yield significant clinical benefits for AD patients, driven by advancements in precision medicine, patient stratification, and tailored medication design.

## Figures and Tables

**Table 1:** Different types of protein kinase, and their function in Alzheimer's disease and other neurogenerative diseases.

**S. No.**	**Protein Kinase**	**Role in Alzheimer's illness**	**Function in Additional Neurodegenerative Conditions**	**References**
1	Tau Kinase	Phosphorylates tau protein, causing neurofibrillary tangles and malfunctioning neurons.	Associated with multiple neurodegenerative disorders, implicated in the process of protein misfolding and clumping together.	[14]
2	GSK-3 (Glycogen Synthase Kinase-3)	Increases the phosphorylation of tau, which contributes to tau pathology and neuronal damage.	Dysregulation is linked to several diseases, including Huntington’s and Parkinson's.	[15]
3	CDK5 (Cyclin-Dependent Kinase 5)	Phosphorylates tau and interferes with synaptic function when activated in AD.	In PD, Huntington's disease, and other disorders, aberrant activation plays a vital role in the degeneration of neurons.	[16]
4	PKA (Protein Kinase A)	alters memory and synaptic plasticity; linked to the etiology of AD.	Variations in neurodegenerative disease expression and activity that affect cognitive function.	[17]
5	MAPK (Mitogen-activated Protein Kinase)	impaired in AD, affecting synaptic plasticity and memory.	Dysregulation of neuronal survival and function linked to several neurodegenerative illnesses.	[18]
6	PI3K/Akt (Phosphoinositide 3-kinase/Akt)	Regulates neuroprotection and cell survival; linked to neurodegenerative illnesses.	Dysregulation resulting from neurodegenerative illnesses, including AD, impacts cell survival and apoptosis.	[19]

**Table 2:** Different types of Protein kinase inhibitors and their mechanism of action in the management of AD.

**S. No.**	**Protein Kinase**	**Drug**	**Mechanism of Action**	**References**
1	GSK-3β (Glycogen Synthase Kinase 3 beta)	LY3200882	GSK-3β inhibitor that is selective and reduces amyloid beta accumulation and tau hyperphosphorylation.	[84]
		Tideglusib	Inhibits GSK-3β action, lowering tau phosphorylation and beta-amyloid formation.	
2	CDK5 (Cyclin-dependent kinase 5)	CP681301	Prevents tau hyperphosphorylation and the development of neurofibrillary tangles by inhibiting CDK5.	[85]
3	MAPK (Mitogen-Activated Protein Kinase)	Xaliproden	Increases neuronal survival and decreases neuroinflammation *via* altering the MAPK pathway.	[86]
4	PKC (Protein Kinase C)	Enzastaurin	PKCβ inhibitor, affecting neuroinflammation and the synthesis of beta-amyloid.	[87]
5	JNK (c-Jun N-terminal Kinase)	T-5224	Reduced oxidative stress and neuronal death by a selective JNK inhibitor.	[88]
6	PI3K (Phosphoinositide 3-kinase)	GNE-317	PI3K inhibitor, changing neural survival pathways and neuroinflammatory responses.	[89]
7	mTOR (Mammalian Target of Rapamycin)	Torin 2	Dual mTORC1/mTORC2 inhibitor that controls synaptic function and autophagy.	[90]
8	CK1 (Casein Kinase 1)	PF-670462	Selective CK1δ/ε inhibitor that affects the phosphorylation and aggregation of tau	[91]
9	CAMKII (Calcium/Calmodulin-Dependent Protein Kinase II)	Autocamtide-2-related inhibitory peptide (AIP)	Inhibiting CAMKII which affects memory formation and synaptic plasticity.	[92]
10	DYRK1A (Dual-Specificity Tyrosine Phosphorylation-Regulated Kinase 1A)	Leucettine L41	A DYRK1A inhibitor affects cognitive decline and synaptic dysfunction.	[93]

## LIST OF ABBREVIATIONS

AD: Alzheimer's Disease
AMPA: Alpha-amino-3-hydroxy-5-methyl-4-isoxazolepropionic acid
APP: Amyloid Precursor Protein
CaMKII: Calcium/calmodulin-Dependent Protein Kinase II
CDK5: Cyclin-Dependent Kinase 5
CMS: Chronic Moderate Stress
Drp1: Dynamin-Related Proteins 1
ERK1/2: Extracellular Regulated Kinase-2
GSK-3: Glycogen Synthase Kinase-3
HSP: Heat Shock Proteins
JNK3-c: Jun N-Terminal Kinase
MOM: Mitochondrial Outer Membrane
mTOR: Mammalian Target of Rapamycin
NFTs: Neurofibrillary Tangles
NMDA: N-Methyl-D-Aspartate
P38 MAPK: Mitogen-Activated Protein Kinase
PKA: Protein Kinase A
SP: Senile Plaques
